# Efficacy and Tolerability of Olaparib Plus Paclitaxel in Patients with Gastric Cancer Associated with Hereditary Breast and Ovarian Cancer

**DOI:** 10.3390/curroncol31110496

**Published:** 2024-10-29

**Authors:** Takuma Hayashi, Kenji Sano, Mako Okada, Manabu Muto, Ikuo Konishi

**Affiliations:** 1 Cancer Medicine, National Hospital Organization Kyoto Medical Center, Fushimi-ku, Kyoto 612-8555, Kyoto, Japan; 2 Pathological Division, Shinshu University Hospital, Matsumoto 390-0877, Nagano, Japan; 3 Medical Oncology, Kyoto University Hospital, Kyoto 612-8555, Kyoto, Japan; 4 Department of Medical Oncology, Kyoto University School of Medicine, Kyoto 606-8507, Kyoto, Japan

**Keywords:** gastric cancer, *Helicobacter pylori*, *BRCA1*, *BRCA2*, hereditary breast and ovarian cancer

## Abstract

*Helicobacter pylori*, a gram-negative, flagellated, helical bacterium, is a common cause of chronic gastric infection worldwide. According to the World Health Organization, *H. pylori* infection, a specific carcinogenic factor, was the leading cause of gastric cancer (GC) in 2014 worldwide (80%). *H. pylori* infection causes GC in >98% of patients in East Asian countries, including Japan. However, only some types of GCs are associated with *H. pylori* infection. Previous clinical studies have revealed that the bacterium secretes cytotoxin-associated gene A antigen, which inhibits the nuclear translocation of the breast cancer susceptibility gene 1 and 2 (*BRCA1/2*), a factor involved in DNA damage repair. This indicated an association between hereditary breast and ovarian cancers (HBOCs) and the development of GC. However, the detailed mechanisms underlying the development of GC caused by *H. pylori* infection remain unclear. Using the information on hereditary cancers obtained based on cancer genomic medicine, this study revealed that the incidence of GC was high in families with HBOC, with a preponderance for men from families with HBOC. Furthermore, the use of poly-adenosine diphosphate-ribose polymerase inhibitors in patients with hereditary GC is considered safe and effective. This study provides substantial evidence for guiding the establishment of early treatment for patients with advanced-stage/metastatic GC who harbored *BRCA1/2* mutations.

## 1. Introduction

Gastric cancer (GC) involves the uncontrolled neoplastic transformation of the mucosal epithelial cells lining the stomach wall that leads to the formation of malignant tumors, which may recur or metastasize [1]. Cancer cells proliferate and gradually invade deeper into the gastric submucosa, muscularis propria, and serosal layers. Eventually, these cancer cells may cross the serosa, spread to the surrounding areas, and invade the surrounding organs, such as the large intestine, pancreas, diaphragm, and liver. In cases of advanced-stage GC, cancer cells can spread beyond the outer serosa and scatter throughout the abdomen; this phenomenon is known as peritoneal dissemination. In addition, distant metastasis can occur via lymphatic or blood flow.

The common symptoms of GC include stomach pain, discomfort, heartburn, nausea, and loss of appetite [2]. Moreover, bleeding may occur in GC tissues, causing anemia and tarry stools. However, these symptoms are also reported by patients with gastritis and/or gastric ulcers. Furthermore, patients with early-stage GC may not experience any symptoms [3], with several patients remaining asymptomatic even after the disease has progressed significantly. Patients with early-stage GC only exhibit a few characteristic subjective symptoms. Thus, it is essential to understand the molecular pathogenesis of GC to develop testing and treatment methods in the initial phases.

Cancer can develop because of the accumulation of multiple cancer-related gene mutations within a single cell. Each cancer type has specific genetic pathogenic variants (PVs) and driver genes. Initially, mutations in the epidermal growth factor receptor (EGFR) gene were identified as the driver of lung cancer. This led to a major paradigm shift in lung cancer treatment [4]. The mutant Kirsten rat sarcoma viral oncogene homolog and mutant *EGFR* act as drivers of various solid cancers, including lung adenocarcinoma and pancreatic cancer [4,5,6]. Similarly, previous clinical studies have shown that *Helicobacter pylori* infection is associated with the development of GC and some malignant lymphomas [7].

*H. pylori* infection causes inflammation and ulcers in the stomach and small intestine. In previous studies, 90% of patients with GC tested positive for *H. pylori* infection. Hence, *H. pylori* infection is a significant risk factor for the development of GC [8,9]. A previous study revealed that *H. pylori* infects gastric mucosal epithelial cells, and cytotoxin-associated gene A (CagA) antigen inhibits the function of DNA damage repair of the breast cancer susceptibility gene (*BRCA1/2*) [10]. Thus, the physiological action of CagA leads to the accumulation of mutations in multiple cancer-related genes within a single cell. However, the molecular mechanism underlying the development of GC caused by *H. pylori* infection remains unclear.

In the United States, the incidence of GC is relatively lower than that of other types of cancer. However, GC is the second most common cause of cancer-related mortality globally. It is quite prevalent in Asian countries such as Korea, China, Taiwan, and Japan, and its treatment usually involves the surgical removal of the cancer lesion, followed by chemotherapy, with or without radiation therapy. In Japan, GC is associated with >50,000 deaths annually [11]. *H. pylori* is a common microbe found in East Asian countries, including Japan [11]. Several patients from East Asia develop GC because of *H. pylori* infection. The bacterium *H. pylori* is broadly classified into CagA-positive and CagA-negative strains based on the presence or absence of CagA secretion, respectively, with the CagA-positive strains causing relatively more severe gastric mucosal lesions [11,12,13,14]. In Western countries, the ratio of CagA-positive–to–CagA-negative strains is 6:4 [13]. However, CagA-positive *H. pylori* strains are more common in East Asian countries such as Japan. Consequently, the incidence of GC in the Japanese population (men = 6.07%, women = 2.11%) (GC characteristic of East Asia) is significantly higher than that in the Western population (USA: men = 0.649%, women = 0.329%; UK: men = 0.6589%, women = 0.279%) [15].

*BRCA1/2* suppresses tumor formation and progression, and recent studies have reported that CagA may prevent the inhibitory effect of *BRCA1/2* on HBOC development [16,17,18,19]. Previous studies have revealed that homologous recombination deficiency (HRD) of several genes, including *BRCA1* and *BRCA2*, may be associated with the development of GC [16,17,18,19]. Injection of *H. pylori* CagA into gastric mucosal epithelial cells significantly increased the accumulation of gene mutations that led to the development of GC [20,21]. Furthermore, the CagA genotype was more likely to have a significant influence on the development of GC [21,22]. Therefore, the action of CagA presumably results in the accumulation of genetic mutations in *BRCA1/2* (i.e., PVs in *BRCA1/2*), which triggers the development and progression of GC by indirectly inducing the cancerous transformation of gastric mucosal epithelial cells. Furthermore, the incidence of GC in patients from families with HBOC harboring germline mutations in *BRCA1/2* may be high [16,17,18,19]. Therefore, this study compared the number of patients with GC from families with and without hereditary breast and ovarian cancer (HBOC) (n = 91 and 94, respectively). Results showed that families with HBOC had a greater proportion of patients with GC than those without [20]. Therefore, treatment with oral poly-adenosine diphosphate-ribose polymerase (PARP) inhibitors such as olaparib, which is an effective drug for patients with platinum-sensitive HBOC or HRD-positive ovarian cancer, can be used in patients with GC harboring *BRCA1/2* mutations with PVs and/or HRD. Furthermore, this study revealed that GC was common in men from families with HBOC. Thus, combination therapy with paclitaxel and platinum drugs or oral treatment with PARP inhibitors, which are effective in patients with platinum-sensitive HBOC or HRD-positive ovarian cancer, can be utilized in patients with GC harboring germline *BRCA1/2* mutations with PVs and/or HRDTo validate the efficacy of olaparib, patients with advanced-stage/metastatic GC in whom PVs were detected in any of the 10 homologous recombination genes (*ATM*, *BARD1*, *BRCA1*, *BRCA2*, *BRIP1*, *CDK12*, *CHEK2*, *PALB2*, *RAD51C*, and *RAD51D*) were randomized to receive either olaparib plus paclitaxel or paclitaxel alone. The safety and efficacy of olaparib plus paclitaxel and paclitaxel alone as a second-line chemotherapy for advanced-stage/metastatic GC were compared. Results showed that the olaparib plus paclitaxel group had a significantly longer overall survival (OS) than the paclitaxel alone group. Hence, large-scale clinical trials should be conducted to further verify the efficacy of olaparib in treating advanced-stage/metastatic GC harboring *BRCA1/2* mutations with PVs or HRD. This study evaluated the results of previous studies [16,17,18,19,20]. Moreover, the use of novel GC treatments based on cancer gene panel testing was explored in the future.

## 2. Patients and Methods

### 2.1. Cancer Gene Panel Profiling

A multicenter, retrospective, observational study was conducted among patients who underwent cancer genomic medicine at various cancer medical facilities in Kyoto, Japan. This clinical study was performed in accordance with the principles of the Declaration of Helsinki and was approved by the Central Ethics Review Board of the National Hospital Organization Headquarters, Meguro, Tokyo, Japan (approval number: NHO R4-04, approval date: 18 November 2020) and the Kyoto University School of Medicine, Kyoto, Japan (approval number: M237, approval date: 24 August 2022). Written informed consent was obtained from all participants.

Cancer genomic medicine was performed using cancer gene panel testing, which was approved by the Japanese Ministry of Health, Labor and Welfare on 3 June 2019. The cancer gene panel testing involved the OncoGuide^TM^ NCC Oncopanel gene mutation analysis set (Sysmex Corporation, Kobe, Hyogo, Japan) and the FoundationOne CDx’s cancer genome test (Foundation One CDx, FoundationOne Liquid CDx; Foundation Medicine, Inc., Cambridge, MA, USA).

Using the FoundationOne CDx cancer genome profile, the mutation information of 324 genes was analyzed in the genomic DNA of tumor tissue samples (including cytology samples) obtained from patients with solid cancer and in the free DNA (cfDNA) of the plasma separated from the whole blood sample of patients with solid cancer. Using the OncoGuideTM NCC Oncopanel, the mutation information of 114 genes was analyzed in the genomic DNA of tumor tissue specimens (including cytology specimens) obtained from patients with solid cancer. In addition, the program analyzed the mutation information of 114 genes in the cfDNA of the plasma separated from the whole blood sample of patients with solid cancer. Furthermore, it determined whether the genomic mutation in the tumor tissue samples was a germline mutation.

HBOC 91 families: total 2424 participants, Patients with gastric cancer 86 patients. Non-HBOC 94 families: total 2377 participants, Patients with gastric cancer 20 patients. From December 2019 to June 2024, 36,211 novel treatments were investigated using cancer genome panel testing (FoundationOne^®^ CDx test: n = 27,981; OncoGuide^TM^ NCC Oncopanel test, Riken Genesis, Yokohama, Kanagawa, Japan, n = 8230) in cancer genomic medicine conducted at Japanese national universities. A novel treatment method was examined using cancer genome panel testing in 2361 Japanese patients with advanced-stage/metastatic GC.

### 2.2. Clinical Trial

In this phase II double-blind clinical study, patients with advanced-stage/metastatic GC were randomized to receive either oral olaparib (100 mg twice per day, tablets) plus intravenous paclitaxel (80 mg/m^2^ per day on days 1, 8, and 15 in each 28-day cycle) or placebo plus paclitaxel (placebo/paclitaxel), followed by maintenance monotherapy with olaparib (200 mg twice per day) or placebo. The clinical study population of patients with germline *BRCA1* and/or *BRCA2* mutations who had PVs and/or HRD was enriched to 50%. The primary endpoints were progression-free survival (PFS) and OS (Supplementary Data).

All patients with GC: total participants n = 132; patients treated with Olaparib + paclitaxel n = 76; patients treated with placebo + paclitaxel n = 65. Patients with HRD-positive GC; total participants n = 68; patients treated with Olaparib + paclitaxel n = 35; patients treated with placebo + paclitaxel n = 33.

### 2.3. Diagnostic Testing for H. pylori Infection

We used highly sensitive tests such as the urea breath test and stool H. pylori antigen test, which should be used in combination with the ABC method. We also stained tissue sections obtained from surgical treatments with hematoxylin and eosin or Giemsa stain and observed them under a microscope. Direct observation allows the detection of H. pylori. Another advantage is that H. pylori infection can be diagnosed even when the bacterium is in the coccoid form (spherical bacteria), which cannot be cultured and has no urease activity.

Of the 86 patients with GC from families with HBOC, 80 (93.02%) had *H. pylori* infection. Of the 20 patients with GC from families without HBOC, 18 (90.00%) presented with *H. pylori* infection. The incidence of *H. pylori* infection was comparable between patients with GC from families with HBOC and those from families without HBOC.

### 2.4. Ethical Considerations for Human Study

Institutional review board (IRB) approval and consent to participate: The current research on human cancer genome information derived from the results of the cancer gene panel testing was conducted at Kyoto University and its affiliated hospitals and the National Hospital Organization Kyoto Medical Center in accordance with institutional guidelines (IRB approval nos.: 50-201504, NHOKMC-2023-2, and H31-cancer-2). This study contains personal and/or medical information and a case report/case history of an identifiable individual. Therefore, data have been sufficiently anonymized in accordance with our anonymization policy. All patients with advanced-stage/metastatic were briefed regarding the clinical study, and they agreed to participate in the current study by providing informed consent.

Ethics committee name: IRB of the National Hospital Organization Headquarters (approval code: H31-cancer-2, approval date: 9 November 2019 and 17 June 2013).

Ethics committee name: IRB of Kyoto University (approval code: R34005, approval date: 1 August 2022).

The authors attended a research ethics education course via the Education for Research Ethics and Integrity (Association for the Promotion of Research Integrity (APRIN) e-learning program [eAPRIN]) agency. The completion numbers for the authors are AP0000151756, AP0000151757, AP0000151769, AP0000151781 and AP000351128.

All data were expressed as the mean and standard error of the mean. The normality of data distribution was validated using the Shapiro–Wilk test. Between-group comparisons were performed using the unpaired two-tailed *t*-test or Mann–Whitney U test. Multiple comparisons were conducted using a one-way analysis of variance with the Tukey post hoc test or the Kruskal–Wallis test with the post hoc Steel–Dwass or Steel test. In general, a *p*-value of <0.05 was considered to indicate statistical significance. All statistical analyses were conducted using JMP software version 15.1 (SAS Institute, Cary, NC, USA).

### 2.5. Data Availability

The data supporting the findings of this clinical study can be obtained from the corresponding author upon reasonable request. The supplementary files available online present the details of the materials and methods used in this study (Study Design, Random Assignment and Masking, Study End Points and Assessments, Statistical Analysis, Management of Nonhematological Treatment-Related AEs Attributed to Olaparib, Management of Nonhematological Treatment-Related AEs Attributed to Paclitaxel).

## 3. Results

### 3.1. Comparison of the Number of Gastric Cancer Cases Between HBOC and Non-HBOC Families

There are limited studies on the combined effects of germline and/or somatic PVs in driver genes and *H. pylori* infection on the development and progression of GC. Results showed that the families with HBOC had a higher incidence of GC (3.55%) than those without (0.78%) (Table 1, Figures S1 and S2). Moreover, men constituted 74.41% of the patients with GC who were from families with HBOC and 55% of the patients with GC who were from families without HBOC (Table 1, Figures S1 and S2).

### 3.2. Detection of Pathogenic Variants in BRCA1 and BRCA2 Genes in Gastric Cancer Tissues by Cancer Genome Panel Testing in a Population of Gastric Cancer Patients

From December 2019 to June 2024, 36,211 novel treatments were investigated using cancer genome panel testing (FoundationOne^®^ CDx test: n = 27,981; OncoGuide^TM^ NCC Oncopanel test, Riken Genesis, Yokohama, Kanagawa, Japan, n = 8230) in cancer genomic medicine conducted at Japanese national universities. A novel treatment method was examined using cancer genome panel testing in 2361 Japanese patients with advanced-stage/metastatic GC. Overall, 2340 (99.11%) of 2361 patients were infected with *H. pylori* based on cancer genome panel testing, and they underwent cancer genome panel testing. Of the 86 patients with GC from families with HBOC, 80 (93.02%) had *H. pylori* infection. Of the 20 patients with GC from families without HBOC, 18 (90.00%) presented with *H. pylori* infection. The incidence of *H. pylori* infection was comparable between patients with GC from families with HBOC and those from families without HBOC. Recent clinical studies have reported that the incidence of *H. pylori* infection in the Japanese population was 5%–10%. In our study with cancer genome panel testing, *BRCA2* with germline PVs (*g*PVs) and/or somatic PVs (*s*PVs) was detected in 395 (395/2361; 16.73%) patients with advanced-stage/metastatic GC. These findings are consistent with histopathological findings in a study using genetically modified Gan mice (Gan*^tgBrca2^*), which showed the reduced progression of epithelial cells in the gastric mucosal tissue of mice [23]. Furthermore, *ERBB2* with PVs was detected in 187 patients with advanced-stage/metastatic GC. The anti-ERBB2 antibody drug may be effective in patients with *ERBB2*-positive advanced-stage GC [23]. The results of our study on cancer genomic medicine are similar to those of a clinical study conducted by He et al. [24].

### 3.3. Antitumor Efficacy and Adverse Events of the PARP Inhibitor Olaparib in Patients with HRD-Positive Gastric Cancer

According to cancer genome panel testing, *BRCA2* with *g*PV and/or *s*PV was detected in 395 (395/2361; 16.73%) of 2361 patients with advanced-stage/metastatic GC. Therefore, treatment with oral PARP inhibitors was initiated in these patients. All patients with advanced-stage/metastatic GC (n = 132) were randomized to receive either olaparib plus paclitaxel (n = 67) or paclitaxel alone (n = 65) (Table 2). In addition, 68 patients with advanced-stage/metastatic GC who had a PV in any of the 10 HRD genes were randomized to receive either olaparib plus paclitaxel (n = 35) or paclitaxel alone (n = 33) (Table 2). To evaluate the suitability of olaparib as a second-line treatment for advanced-stage/metastatic GC, the efficacy and safety of olaparib plus paclitaxel and paclitaxel alone were compared. Results showed that the olaparib plus paclitaxel group had a significantly longer PFS than the paclitaxel alone group (all patients: hazard ratio [HR] = 0.81, 95% confidence interval [CI] = 0.61–1.04, *p* = 0.032; patients with GC who presented with HRD: HR = 0.61, 95% CI = 0.52–1.11, *p* = 0.067). Similarly, the olaparib plus paclitaxel group had a significantly longer OS than the paclitaxel alone group (all patients: HR = 0.57, 95% CI = 0.34–0.86, *p* = 0.010; patients with GC who presented with HRD: HR = 0.36, 95% CI = 0.16–0.72, *p* = 0.003).

Table 3 shows the most commonly observed adverse events (AEs) observed in the study patients. The proportion of patients experiencing AEs was similar between the two groups, and the AEs was classified as ≥grade 3 as per the Common Terminology Criteria for AEs (all patients: olaparib/paclitaxel [n = 47, 70.1%], placebo/paclitaxel [n = 48, 73.8%]; patients with GC presenting with HRDs: olaparib/paclitaxel [n = 25, 71.4%], placebo/paclitaxel [n = 23, 70.0%]). The placebo/paclitaxel arm had a higher proportion of patients with serious AEs (SAEs) than the olaparib/paclitaxel arm. The most common SAE was pneumonia (all patients: olaparib/paclitaxel [n = 4, 5.9%], placebo/paclitaxel [n = 6, 9.2%]; patients with GC who presented with HRDs: olaparib/paclitaxel [n = 2, 5.7%], placebo/paclitaxel [n = 2, 6.1%]). None of the chemical parameters changed significantly. The AEs observed were consistent with the known profile of paclitaxel. None of the patients exhibited unexpected safety signs or clinically important changes in vital signs, electrocardiogram parameters, or physical examination results during the study. No SAEs with a mortality outcome that can be causally related to study treatment (according to the investigator’s assessment) were reported.

## 4. Discussion

The American Cancer Society has reported that approximately 24,590 Americans will be diagnosed with GC annually, and 10,720 Americans will die from the disease [25]. The 5-year survival rate of patients with GC (all stages combined) is approximately 28% [25]. BRCA1 and BRCA2 are proteins involved in DNA damage repair and are expressed in all cells. In particular, as these proteins suppress the neoplastic transformation of normal cells, an association was observed between HBOC and the development of GC. However, the detailed mechanism by which *H. pylori* infection induces the development of GC remains unclear. Using the information on hereditary cancers obtained based on cancer genomic medicine conducted at university hospitals in Japan, our study revealed that the incidence of GC in families with HBOC was high. In families with HBOC, the incidence of GC was slightly higher in men than in women. Furthermore, treatment with PARP inhibitors may be effective in patients with hereditary advanced-stage/metastatic GC.

*H. pylori* infects both men and women without significant differences in the infection rate between the two sexes. However, compared with noninfected people, the probabilities of developing GC from birth to 85 years of age in patients with *H. pylori* infection are 17.0% (approximately 1 in 6 people) for men and 7.7% (approximately 1 in 13 people) for women [26]. Men have a higher incidence of GC caused by *H. pylori* infection than women. Presumably, these sex-related differences observed in our study can be attributed to the relatively higher number of men with GC observed in 91 families with HBOC and 94 families without HBOC (74.41% vs. 55.00%; Table 1). Nevertheless, the reason for sex-based differences in the incidence of GC in families with HBOC remains unclear. Further clinical studies must be conducted to validate these reasons.

A Korean research group showed that GC cell lines, particularly those with significantly low levels of ataxia telangiectasia (a key activating gene in the HR gene family) mutations (ATM), were sensitive to PARP inhibitors such as olaparib, niraparib, and talazoparib [27]. The research group compared the efficacy of olaparib plus paclitaxel (olaparib/paclitaxel) versus paclitaxel alone (placebo/paclitaxel) in patients with advanced-stage/metastatic GC and evaluated whether a low ATM expression was a predictor of improved clinical outcomes in patients treated with olaparib/paclitaxel. Results showed that the combination therapy was more effective in treating advanced-stage/metastatic GC and was associated with a prolonged OS in patients with low levels of ATM [27].

Previous clinical trials have investigated the efficacy of olaparib in patients with GC who had extremely low or no ATM expression [27]. Therefore, patients with GC were categorized into cohorts with positive or negative expression of ATM in GC tissue. A clinical trial conducted by our medical team investigated the efficacy of olaparib in patients with advanced-stage/metastatic GC who had *g*BRCA1 with PVs, *g*BRCA2 with PVs, and/or HRD. Therefore, patients with GC were categorized into cohorts with and without *g*BRCA1 PVs, *g*BRCA2 PVs, or HRD in GC tissue. The clinical trial protocol conducted by our medical staff was different from the protocols that have already been published.

Another Japanese research group revealed that germline pathogenic mutations in the nine genes of the HR gene family (*APC*, *ATM*, *BRCA1*, *BRCA2*, *CDH1*, *MLH1*, *MSH2*, *MSH6*, and *PALB2*) are associated with the risk of GC in Japanese patients treated at the Aichi Cancer Center Hospital Epidemiology Research Program, Nagoya, Achi, Japan [17,27,28,29,30]. They found a significant association between *H. pylori* infection and these pathogenic mutations in the HR gene in patients with advanced-stage/metastatic GC.

HBOC syndrome is a cancer susceptibility syndrome caused by germline mutations in *BRCA1* and/or *BRCA2* and is inherited in an autosomal dominant manner [31]. Previous studies have identified other candidate genes, in addition to HBOC causative genes, that specifically contribute to the HBOC onset. For example, genes involved in DNA damage checkpoint, such as *ATM* and *CHEK2,* and those involved in the repair of DNA double-strand breaks, such as *PALB2*, *BRIP1*, and *RAD51C,* contribute to HBOC onset [32,33,34,35,36]. Previous clinical studies have shown that PVs in these genes are closely associated with the development of breast, ovarian, prostate, or pancreatic cancers. Furthermore, carriers of *PALB2* mutation have a 35% cumulative risk of developing breast cancer at the age of 70 years [37], and ATM with PVs has been detected in 1.2%–6.9% of all breast cancer cases [37].

Based on the results of previous clinical studies, 10 genes in the HR gene family, which are associated with the development of breast, ovarian, prostate, and pancreatic cancers, were significantly involved in the development of GC. Therefore, our research team conducted a comparative study of the incidence of GC in patients from families with HBOC and those from families without HBOC. Results showed that the incidence of GC was higher in patients from families with HBOC than in those from families without HBOC (3.55% vs. 0.78%) (Table 1, Figures S1 and S2). PARP inhibitors are effective in treating patients with ovarian, breast, prostate, or pancreatic cancers who had HRD and/or *BRCA1/2* mutations with PVs. Therefore, all patients with GC were randomized to receive either olaparib plus paclitaxel (n = 67) or paclitaxel alone (n = 65). Furthermore, patients with GC from families with HBOC were randomized to receive olaparib plus paclitaxel (n = 35) or paclitaxel alone (n = 33). Patients with GC from families with HBOC who received combination therapy had prolonged PFS and OS compared with those who were treated with paclitaxel alone. However, a previous clinical study did not show significant improvements in the OS of the overall or *ATM*-negative population of Asian patients with advanced-stage GC who received olaparib [38,39]. Therefore, combination therapy with olaparib plus paclitaxel can be used as a second-line treatment for patients with advanced-stage/recurrent GC who had HRD and/or *BRCA1/2* mutations with PVs. Further clinical trials should be performed to establish the optimal regimen for combination therapy with olaparib plus paclitaxel in this population.

The Japanese Ministry of Health, Labor, and Welfare has approved insurance coverage for treatment with oral PARP inhibitors such as olaparib and niraparib. Olaparib has been approved for patients with breast, ovarian, pancreatic, or prostate cancer who had HRD or *BRCA1/2* mutations with PVs, whereas niraparib has been approved for patients with ovarian cancer who had HRD or *BRCA1/2* mutations with PVs. The suitability was determined using the MyChoice test (South San Francisco, Myriad Genetics, Inc., CA, USA). In addition, the insurance coverage was approved for the oral administration of talazoparib in patients with breast cancer who had *BRCA1/2* mutations with PVs detected via BRACanalysis and those with prostate cancer who had *BRCA1/2* mutations with PVs detected using FoundationOne CDx. Based on the available clinical evidence, oral treatment with PARP inhibitors can be considered a treatment modality for patients with GC who had HRD and/or *BRCA1/2* mutations with PVs. Our research findings offer substantial evidence for guiding the establishment of early treatment for patients with advanced-stage/metastatic GC who had *BRCA1/2* mutations with PVs. This study provides useful data on the efficacy and safety of olaparib plus paclitaxel, a chemotherapeutic agent, and a basis for future studies in this patient population, which is challenging to manage. The clinical trials conducted to date involve small cohorts. Thus, further studies with larger cohorts are warranted to validate the efficacy of PARP inhibitors in treating patients with advanced-stage/metastatic GC who had HRD and/or *BRCA1/2* mutations with PVs.

## Figures and Tables

**Table 1:** Distribution of patients with hereditary GC from families with HBOC reflecting the extent of genetic testing ^1^.

	Number of Patients with GC from Families with HBOC (Families n = 91)		Number of Patients with GC from Families Without HBOC (Families n = 94)	
Generation	Patients with GC ^2^ (Other Types of Tumors ^3^)	Incidence (%) of GC ^2^ (Total Number of Patients)	Patients with GC ^2^ (Other Types of Tumors ^3^)	Incidence (%) of GC ^2^ (Total Number of Patients)
I	15 (26)	4.36% (344)	1 (6)	0.31% (358)
II	47 (197)	6.18% (761)	6 (45)	0.74% (730)
III	19 (145)	3.24% (587)	11 (42)	1.73% (572)
IV	4 (12)	0.99% (405)	2 (7)	0.55% (395)
V	1 (1)	033% (299)	0 (2)	0.00% (281)
VI	0 (0)	0.00% (28)	0 (0)	0.00% (41)
Total cases	86 (381)	3.55% (2424)	20 (102)	0.84% (2377)
Sex-wise distribution of patients with GC				
Total cases	Patients with GC from families with HBOC (n = 86)		Patients with GC from families without HBOC (n = 20)	
	64 men with GC	74.41% men	11 men with GC	55.0% men with GC

Genetic testing ^1^: BRACAnalysis^®^ Diagnostic System (Myriad Genetics G.K., Zurich, Switzerland), GC ^2^: gastric cancer, and other types of tumors ^3^: HBOC-related cancers (hereditary cancers including breast cancer, ovarian cancer, prostate cancer, and pancreatic cancer).

**Table 2:** Clinical effects of combination therapy with olaparib plus paclitaxel in patients with HRD-positive GC. Best objective response (evaluable-for-response analysis set).

	All Patients with GC		Patients with HRD-Positive GC *	
	Olaparib + PTX Group	Placebo + PTX Group	Olaparib + PTX Group	Placebo + PTX Group
Total number of cases	n = 67	n = 65	n = 35	n = 33
Median PFS (months)	4.92	3.25	5.39	3.59
HR	0.81 95% CI: 0.61–1.04, *p* = 0.032		0.61 95% CI: 0.52–1.11, *p* = 0.067	
Median OS (months)	13.1	8.3	19.5	8.2
HR	0.57 95% CI: 0.34–0.86, *p* = 0.010		0.36 95% CI: 0.16–0.72, *p* = 0.003	

CI: confidence interval; GC: gastric cancer; HRD: homologous recombination deficiency; PTX: paclitaxel; PFS: progression-free survival; HR: hazard ratio; OS: overall survival. HRD-positive GC cases * include patients with advanced-stage/metastatic GC with HRD and/or *BRCA1/2* mutations with pathological variants.

**Table 3:** AEs (any grade) in 20% of all patients or ≥grade 3 AEs in 5% of all patients (arranged by the MedDRA Preferred Term).

	No. of Patients (%)							
	All Patients with GC				Patients with HRD-Positive GC			
	Olaparib/Paclitaxel Group (n = 67)		Placebo/Paclitaxel Group (n = 65)		Olaparib/Paclitaxel Group (n = 35)		Placebo/Paclitaxel Group (n = 33)	
AEs	Any Grade	>Grade 3	Any Grade	>Grade 3	Any Grade	>Grade 3	Any Grade	>Grade 3
Hematologic AEs								
Anemia	12 (18)	7 (11)	12 (19)	7 (11)	6 (17)	3 (9)	5 (17)	3 (9)
Neutropenia	50 (75)	31 (46)	40 (65)	25 (39)	26 (74)	6 (17)	(70)	7 (21)
Nonhematologic AEs								
Alopecia	31 (46)	0	31 (47)	0	15 (43)	0	23 (40)	0
Decreased appetite	23 (34)	0	27 (42)	0	11 (32)	0	5 (31)	0
Peripheral neuropathy	24 (36)	2 (3)	14 (21)	1 (2)	10 (31)	0	8 (23)	0
Nausea	21 (31)	0	26 (40)	0	9 (26)	8 (22)	7 (22)	6 (18)
Asthenia	20 (30)	2 (3)	18 (27)	7 (10)	10 (28)	0	10 (20)	0
Diarrhea	21 (31)	2 (3)	18 (27)	1 (2)	11 (29)	2 (6)	7 (25)	0
Abdominal pain	15 (22)	0	16 (24)	2 (3)	3 (21)	0	1 (18)	0
Fatigue	16 (24)	1 (10.4)	21 (32)	2 (3)	3 (23)	3 (9)	6 (23)	3 (9)
Myalgia	10 (15)	0	23 (36)	0	7 (13)	2 (6)	12 (36)	3 (9)
Pneumonia	4 (6)	2 (3)	6 (9)	3 (5)	2 (6)	1 (3)	2 (6)	1 (3)

AE: adverse event; GC: gastric cancer; HRD: homologous recombination deficiency; MedDRA: Medical Dictionary for Regulatory Activities. In addition to neutropenia, two patients experienced febrile neutropenia. The sentence is as follows: Both events occurred in the olaparib/paclitaxel arm during the combination phase; one event was grade 3 by Common Terminology Criteria for Adverse Events (the other was grade 2) and led to dose modification. Patients with HRD-positive GC refer to patients with advanced-stage/metastatic GC with HRD and/or *BRCA1/2* mutations who had PVs.

## Data Availability

Data are available on various websites and have also been made publicly available. More information can be found in the first paragraph of the Results section. The transparency document associated with this article can be found in the online version at https://kyoto.hosp.go.jp/html/guide/medicalinfo/clinicalresearch/expand/gan.html (accessed on 18 May 2024).

## Supplementary Materials

The following supporting information can be downloaded at: https://www.mdpi.com/article/10.3390/curroncol31110496/s1, Figure S1: HBOC family; Figure S2: Non-HBOC family; File S1: Study Design.

## Abbreviations

AE: adverse event, BRCA: breast cancer susceptibility gene, CagA: cytotoxin-associated gene A antigen, GC: gastric cancer, HBOC: hereditary breast and ovarian cancer, *H. pylori*: *Helicobacter pylori*, HRD: homologous recombination deficiency, OS: overall survival, PFS: progression-free survival, PARP: poly-adenosine diphosphate-ribose polymerase, WHO: World Health Organization.
